# Effect of hypobaria on maximal ventilation, oxygen uptake, and exercise performance during running under hypobaric normoxic conditions

**DOI:** 10.14814/phy2.14002

**Published:** 2019-02-12

**Authors:** Takeshi Ogawa, Naoto Fujii, Yasuhiro Kurimoto, Takeshi Nishiyasu

**Affiliations:** ^1^ Department of Physical Education Osaka Kyoiku University Kashiwara Osaka Japan; ^2^ Faculty of Health and Sports Science University of Tsukuba Tsukuba Japan

**Keywords:** Airflow resistance, altitude, hypobaric condition, ventilation, $\overset{\cdot }{V}$O_2max_

## Abstract

During exposure to high altitude, hypoxia develops because of reductions in barometric pressure and partial pressure of O_2_. Although several studies have examined the effects of hypoxia on exercise performance and physiological responses, such as maximal minute ventilation ($\overset{\cdot }{V}$_Emax_) and maximal oxygen uptake ($\overset{\cdot }{V}$O_2max_), how barometric pressure reduction (hypobaria) modulates them remains largely unknown. In this study, 11 young men performed incremental treadmill running tests to exhaustion under three conditions chosen at random: normobaric normoxia (NN; 763 ± 5 mmHg of barometric pressure, equivalent to sea level), hypobaric hypoxia (HH; 492 ± 1 mmHg of barometric pressure, equivalent to 3500 m above sea level (m a.s.l.)), and hypobaric normoxia (HN; 492 ± 1 mmHg of barometric pressure while breathing 32.2 ± 0.1% O_2_ to match the inspiratory O_2_ content under NN). $\overset{\cdot }{V}$_Emax_ was higher in HN than in NN (160.9 ± 10.7 vs. 150.7 ± 10.0 L min^−1^, *P* < 0.05). However, no differences in $\overset{\cdot }{V}$O_2max_ and arterial oxyhemoglobin saturation were observed between NN and HN (all *P* > 0.05). Time to exhaustion was longer in HN than in NN (932 ± 83 vs. 910 ± 79 s, *P* < 0.05). These results suggest that reduced air density during exposure to an altitude of 3500 m a.s.l. increases maximal ventilation and extends time to exhaustion without affecting oxygen consumption or arterial oxygen saturation.

## Introduction

Maximal oxygen uptake ($\overset{\cdot }{V}$O_2max_) and endurance exercise performance decline with elevations in altitude because of reduced ambient partial O_2_ pressure (Fulco et al. 1998; Derchak et al. 2000). Pulmonary ventilation increases exponentially with decreases in ambient partial O_2_ pressure. This response partly counteracts reduced alveolar partial pressures of oxygen (P_A_O_2_) and thus, arterial oxyhemoglobin saturation (SaO_2_) (Calbet et al. 2003; Ogawa et al. 2007). Previous studies have demonstrated that individuals with greater increases in maximal minute ventilation ($\overset{\cdot }{V}$E_max_) under acute hypobaric hypoxia (HH) relative to normobaric normoxia (NN) showed smaller reductions in $\overset{\cdot }{V}$O_2max_ (Marconi et al. 2004; Ogawa et al. 2007). Therefore, greater increases in ventilation during hypoxic exercise appear to be beneficial for minimizing reductions in $\overset{\cdot }{V}$O_2max_.

In most acute hypoxia studies, normobaric hypoxia (NH) condition is employed to investigate the influences of exposure to high altitude on physiological responses and exercise performance. However, whether HH and NH are physiologically equivalent remains debatable (Millet et al. 2012). For example, resting $\overset{\cdot }{V}$_E_ and SaO_2_ tend to be lower under HH conditions than under NH conditions (Coppel et al. 2015). Furthermore, Saugy et al. (2016) showed that the magnitude of the reduction in cycling performance was greater during exposures to HH compared to that with NH exposure (Coppel et al. 2015), which implied that the HH condition might be more detrimental to exercise performance and physiological responses.

Under HH condition (e.g., high‐altitude exposure), air density, and therefore, air resistance, are lower than they are at sea level (Gautier et al. 1997). Thus, reductions in barometric pressure that are associated with acute high‐altitude exposures could affect the physiological responses. Studies have demonstrated that breathing a helium–oxygen (He–O_2_) gas mixture, which could greatly reduce airflow resistance (Mink and Wood 1980; Papamoschou 1995), increases $\overset{\cdot }{V}$_Emax_ during maximal exercise under hypoxic conditions relative to breathing non‐He–O_2_ under controlled conditions. Moreover, increases in $\overset{\cdot }{V}$O_2max_ and $\overset{\cdot }{V}$_Emax_ were observed by breathing He–O_2_ compared to that with non‐He–O_2_ (Esposito and Ferretti 1997; Ogawa et al. 2010), even under the NN condition (Powers et al. 1986). Furthermore, the effect of hypobaric normoxia (HN) was explored in early studies (Cerretelli 1976; Marconi et al. 2004) of chronic high‐altitude conditions with pure enriched O_2_ gas mixture breathing. Those studies showed that $\overset{\cdot }{V}$O_2max_ was higher in HN than in NN. Whether reduced air density in acute hypobaric conditions increases $\overset{\cdot }{V}$_Emax_ and $\overset{\cdot }{V}$O_2max_ in a similar manner to that observed with He–O_2_ breathing and chronic HN remains to be determined.

Therefore, this study tested the hypothesis that acute hypobaria associated with exposure to the HH condition increases $\overset{\cdot }{V}$_E_ and $\overset{\cdot }{V}$O_2max_, thereby improving endurance exercise performance. Further, as a secondary purpose, we estimated whether hypobaria would lower the oxygen consumption of respiratory muscles. If reduced air density under hypobaric conditions could lower $\overset{\cdot }{V}$O_2_ in the respiratory muscles due to the decreased work of breathing, this might improve exercise performance. Similarly, Harms et al. (1997, 1998, 2000) reported that unloading the respiratory muscles’ work during intensive exercise resulted in a greater distribution of the available cardiac output to the active locomotor muscles, thereby improving exercise tolerance with no change in $\overset{\cdot }{V}$O_2_.

## Materials and Methods

### Ethical approval

This study was approved by the Human Subjects Committees of the University of Tsukuba in accordance with the guidelines set forth in the Declaration of Helsinki. All participants provided verbal and written informed consent before participating in this study.

### Participants

Eleven healthy young men (age, 24 ± 4 years; height, 1.73 ± 0.07 m; body mass, 63.3 ± 4.8 kg) including three physically active students and eight long‐ or middle‐distance runners on the university track and field team participated in this study. All participants lived at low altitudes and had not been exposed to altitudes >1000 m within the 6 months prior to the study.

### Incremental running test

Each participant performed an incremental running test to exhaustion in an environmental chamber (Shimazu Co. Ltd., Kyoto, Japan) under three conditions (performed randomly and on separate days): NN (20.9 ± 0.1% O_2_ at 763 ± 5 mmHg of barometric pressure, equivalent to sea level), HH (20.9 ± 0.1% O_2_ at 492 ± 1 mmHg of barometric pressure, equivalent to 3500 meters above sea level (m a.s.l.)), and hypobaric normoxia (HN; 32.2 ± 0.1% O_2_ at 492 ± 1 mmHg of barometric pressure). The partial pressure of O_2_ in NN and HN were matched (159 mmHg in both conditions), which enabled an assessment of the effects of reducing the barometric pressure without stimulating a hypoxic effect. The study room temperature was maintained at 20.2 ± 0.4°C and was continuously ventilated to minimize increases in the CO_2_ concentration in the air. Each participant performed self‐selected warm‐up exercises (stretching and jogging) outside the laboratory. The structure of the warm‐up was similar in all three conditions. Thereafter, participants entered the environmental chamber. For the hypobaric conditions (i.e., HH and HN), the chamber was gradually decompressed to achieve a barometric pressure equivalent to that at 3500 m a.s.l. in 20 min. For safety reasons, we avoided rapid decompression of the chamber. Each running test began within 20 min after completing the decompression. Under all conditions, the participants breathed through a face mask that covered the nose and mouth. The mask was connected via low‐resistance silicon pipes to a large reservoir bag. The incremental running test was performed on a treadmill at an inclination of 0°, which was maintained throughout the experiment. The initial running speed was set at 160 to 220 m/min, depending on the participant's running ability and was subsequently increased by 20 m/min every 2 min, such that 240 or 280 m/min was achieved within 15 min. Thereafter, the running speed was increased by 10 m/min every 1 min until exhaustion (Ogawa et al. 2007, 2010). When nearing $\overset{\cdot }{V}$O_2max_, the expired gas was collected in Douglas reservoir bags every 1 min.

### Mimic ventilation trial

As a secondary test, 10 of the 11 participants who completed the incremental running test subsequently participated in a mimic ventilation trial performed under NN and HH conditions (in random order) to determine the oxygen consumption of the respiratory muscles during the incremental running test. After obtaining 5‐min baseline resting measurements in either NN or HH, the participants started a voluntary hyperventilation process while in the standing position. Since SaO_2_ was 100% under HH conditions during the voluntary hyperventilation process, HH under the mimic ventilation trial was assumed to be the same as HN. The participants were instructed to reproduce the tidal volume (VT) and respiratory frequency (*f*_R_) observed at $\overset{\cdot }{V}$O_2max_ under each condition for 7 min. VT and *f*_R_ were adjusted to the target level using a computer that showed breath‐by‐breath measurements of VT and *f*_R_. During the mimic ventilation, 100% CO_2_ was added to the inspiratory gas to maintain the end tidal pressure of CO_2_ (P_ET_CO_2_) at normocapnic levels.

### Measurements

#### Incremental running test

$\overset{\cdot }{V}$O_2_, $\overset{\cdot }{V}$CO_2_, and $\overset{\cdot }{V}$_E_ were calculated using the Douglas bag method. O_2_ and CO_2_ concentrations were measured using a mass spectrometer (ARCO1000; ARCO; Chiba, Japan), which was calibrated with a standardized gas of known composition (O_2_, 15.00%; CO_2_, 5.00%; and N_2_, balanced). The volume inside the bag was determined using a dry gas meter (DC‐5A; Shinagawa; Tokyo, Japan), which was carefully calibrated with a 2‐L syringe before the experiment. All participants accomplished two of the following three criteria for $\overset{\cdot }{V}$O_2max_: constant $\overset{\cdot }{V}$O_2_ despite increases in running speed (increase in <2.0 mL kg^−1^ min^−1^); the respiratory quotient >1.1; maximal heart rate (HRmax) achieved was >90% of the age‐predicted value. Moreover, all participants reported a Borg scale of 20 and were not able to maintain the last‐stage running speed despite strong verbal encouragement. We also measured expiratory O_2_ and CO_2_ fractions (F_E_O_2_ and F_E_CO_2_) breath‐by‐breath using a mass spectrometer (ARCO1000). We estimated P_A_O_2_ as:

$${\text{PAO}}_{2}={\text{PIO}}_{2}-\left({\text{PETO}}_{2}/R\right),$$

where P_I_O_2_ is the partial pressure of inspiratory O_2_, P_ET_O_2_ is the end tidal O_2_ pressure, and R is the respiratory quotient.

Alveolar ventilation ($\overset{\cdot }{V}$_A_) was calculated as:

$$\overset{\cdot }{\underset{A}{V}}=\left(V^{\cdot }O_{2}\times R\times 0.863\right)/P_{\mathrm{ET}}{\mathrm{CO}}_{2},$$

where P_ET_CO_2_ is the end tidal CO_2_ pressure. SaO_2_ and heart rate (HR) were measured using a forehead pulse oximeter (N‐595; Nellcor, Hayward, CA) and an HR monitor (Vantage NV; POLAR, Finland), respectively. In this study, time to exhaustion during incremental testing was used as an index of exercise performance.

#### Mimic ventilatory test

Breath‐by‐breath F_E_O_2_ and F_E_CO_2_ and flow volume were determined using a mass spectrometer (ARCO1000) and a spirometer (MINATO AS300i; Minato Medical; Osaka, Japan), respectively. During voluntary hyperventilation, $\overset{\cdot }{V}$O_2_, $\overset{\cdot }{V}$_E_, VT, and *f*_R_ were calculated. The $\overset{\cdot }{V}$O_2_ of respiratory muscles ($\overset{\cdot }{V}$O_2rm_) was calculated by subtracting resting $\overset{\cdot }{V}$O_2_ from $\overset{\cdot }{V}$O_2_ recorded during the last 30 s of voluntary hyperventilation ($\overset{\cdot }{V}$O_2vent_). The mouth pressure was measured using a pressure transducer probe inserted into a mouthpiece and was reported at a sampling rate of 200 Hz. The peak inspiratory and expiratory mouth pressures (P_Imax_ and P_Emax_) were determined during each respiratory cycle.

### Statistical analysis

Data are expressed as means ± standard deviations (SD). Variables obtained during the incremental exercise tests were analyzed using one‐way repeated‐measures analyses of variance with an experimental condition factor (NN, HH, and HN). After detecting the main effects, Fisher's least significant difference tests were performed as post hoc tests. Variables obtained during the mimic ventilatory test were analyzed using paired t‐tests (NN vs. HN). *P* values < 0.05 were considered statistically significant. SPSS 24 (IBM Corp., Armonk, NY) was used for all statistical analyses.

## Results

### Incremental running test

Resting SaO_2_ was similar for HN versus NN (98 ± 2% vs. 98 ± 2%). $\overset{\cdot }{V}$_Emax_ was 6.8% higher in HN than in NN (Table 1). As hypothesized, $\overset{\cdot }{V}$_Emax_ was higher (4.3%) in HN than in HH (Table 1). Similarly, *f*_R_ was higher in HN than in HH (Table 1). Greater ventilation was not paralleled by greater $\overset{\cdot }{V}$O_2max_ such that $\overset{\cdot }{V}$O_2max_ was similar between HN and NN (Table 1). However, the time to exhaustion was longer in HN than in NN (Table 1). No difference in $\overset{\cdot }{V}$CO_2max_ between NN and HN was noted. SaO_2_ at the point of exhaustion did not differ between HH and HN and no difference in maximal HR_max_ between NN and HN was observed (Table 1).

**Table 1:** Variables measured at $\overset{\cdot }{V}$O_2max_

	NN	HH	HN
$\overset{\cdot }{V}$O_2max_ [mL min^−1^]	3974 ± 338	2860 ± 241^a^	4011 ± 327^b^
$\overset{\cdot }{V}$O_2max_ [mL kg^−1^ min^−1^]	63.0 ± 4.7	46.0 ± 5.6^a^	63.6 ± 5.6^b^
$\overset{\cdot }{V}$CO_2max_ [mL min^−1^]	4580 ± 282	3506 ± 215^a^	4531 ± 327^b^
$\overset{\cdot }{V}$_Emax_ [L min^−1^]	150.7 ± 10.0	154.2 ± 11.8	160.9 ± 10.6^a^, ^b^
*f*_R_ [breaths min^−1^]	68 ± 10	70 ± 10	73 ± 10^a^, ^b^
VT [L]	2.2 ± 1.03	2.25 ± 0.36	2.25 ± 0.3
$\overset{\cdot }{V}$_E_ $\overset{\cdot }{V}$O_2_^−1^ [ml ml^−1^]	38.1 ± 3.6	54.3 ± 6.6^a^	40.4 ± 4.9^a^, ^b^
$\overset{\cdot }{V}$_E_ $\overset{\cdot }{V}$CO_2_^−1^ [ml ml^−1^]	33.0 ± 3.1	44.0 ± 3.1^a^	35.6 ± 3.5^a^, ^b^
$\overset{\cdot }{V}$_A_ [L min^−1^]	101.8 ± 6.6	95.3 ± 10.0^a^	106.8 ± 8.6^b^
P_ET_CO_2_ [mmHg]	39.2 ± 3.3	31.6 ± 3.9^a^	36.8 ± 3.5^b^
P_A_O_2_ [mmHg]	128.5 ± 10.6	76.9 ± 3.5^a^	126.2 ± 4.2^b^
SaO_2_ [%]	91 ± 3	69 ± 4^a^	90 ± 5^b^
HR_max_ [beats min^−1^]	195 ± 3	181 ± 8^a^	192 ± 8^b^
Time to exhaustion [s]	910 ± 79	614 ± 73^a^	932 ± 83^a^, ^b^

Values are mean ± standard deviation (*n* = 11).

NN: normobaric normoxia; HH: hypobaric hypoxia; HN: hypobaric normoxia; $\overset{\cdot }{V}$O_2max_: maximal oxygen uptake; $\overset{\cdot }{V}$CO_2max_: maximal carbon dioxide output; *f*_R_: respiratory frequency; VT: tidal volume; V_A_: alveolar ventilation; P_ET_CO_2_: end tidal CO_2_ pressure; P_A_O_2_: partial pressure of alveolar O_2_; SaO_2_: arterial oxyhemoglobin saturation; HR_max_: maximal heart rate.

a *P* < 0.05 versus NN.

b *P* < 0.05 versus HH.

### Mimic ventilation trial

**Figure 1:**
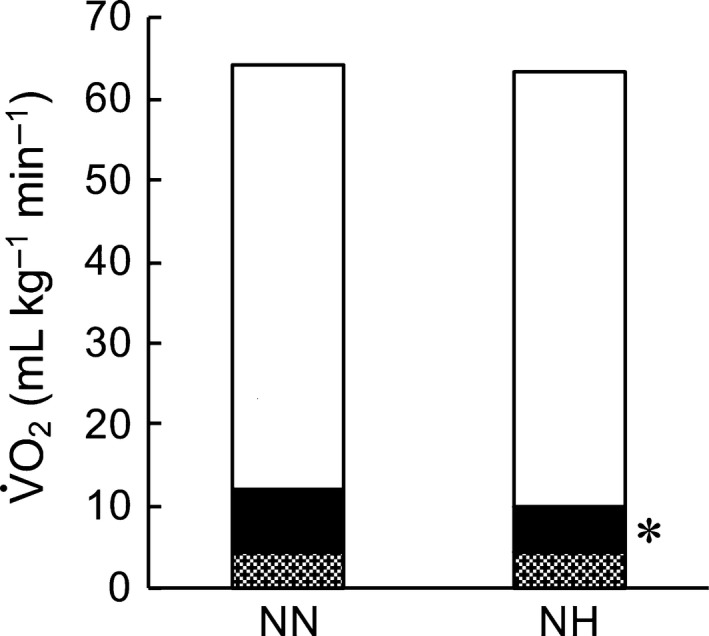
Estimated oxygen consumption at $\overset{\cdot }{V}$O_2max_. Grey area shows $\overset{\cdot }{V}$O_2_ at rest, the black area shows the $\overset{\cdot }{V}$O_2_ of the respiratory muscles, and the white area shows other tissues. NN, normobaric normoxia; HN, hypobaric normoxia. *P < 0.05 versus NN.

Table 2 and Figure 1 show the results of the mimic ventilation trials. The participants controlled their VT and *f*_R_ to achieve the level of $\overset{\cdot }{V}$_Emax_ in NN and HN. SaO_2_ was 100 ± 0% in both HN and NN conditions. $\overset{\cdot }{V}$O_2vent_ was lower in HN than in NN. $\overset{\cdot }{V}$O_2rm_ was 23.1% lower in HN than in NN (5.7 ± 1.8 vs. 7.7 ± 2.0 mL kg^−1^ min^−1^, respectively; *P* < 0.05). Thus, the calculated percentage of $\overset{\cdot }{V}$O_2rm_ against whole‐body $\overset{\cdot }{V}$O_2max_ was lower in HN than in NN (9.1 ± 3.4 vs. 12.4 ± 3.6%, *P* < 0.05). P_Imax_ was 27.6% lower in HN than in NN. P_Emax_ was 23.2% lower in HH than in NN (Table 2).

**Table 2:** Variables analyzed during the last 30 s of voluntary hyperventilation at rest

	NN	HN	% change
$\overset{\cdot }{V}$_E_ [L min^−1^]	147.9 ± 11.9	158.1 ± 12.7^a^	6.9
*f*_R_ [breaths min^−1^]	68 ± 10	73 ± 11^a^	7.4
VT [L]	2.23 ± 0.28	2.24 ± 0.3	−0.2
$\overset{\cdot }{V}$O_2mimc_ [mL kg^−1^ min^−1^]	12.19 ± 2.11	10.15 ± 1.66^a^	−15.5
$\overset{\cdot }{V}$O_2rest_ [mL kg^−1^ min^−1^]	4.46 ± 0.84	4.48 ± 1.02	1.11
$\overset{\cdot }{V}$O_2vent_ [mL kg^−1^ min^−1^]	7.73 ± 2.04	5.67 ± 1.80^a^	−23.1
%$\overset{\cdot }{V}$O_2max_	12.4 ± 3.6	9.1 ± 3.4^a^	−23.4
SaO_2_ [%]	100 ± 0	100 ± 0	0
P_Imax_ [cmH_2_O]	8.98 ± 2.80	6.20 ± 2.00^a^	−27.6
P_Emax_ [cmH_2_O]	−9.15 ± 2.11	−6.87 ± 1.59^a^	−23.2

Values are mean ± standard deviation (*n* = 10).

NN: normobaric normoxia; HN: hypobaric normoxia; $\overset{\cdot }{V}$_E_: minute ventilation; *f*_R_: respiratory frequency; VT: tidal volume; $\overset{\cdot }{V}$O_2mimc_: oxygen uptake during mimic ventilation; $\overset{\cdot }{V}$O_2rest_: oxygen uptake at rest; $\overset{\cdot }{V}$O_2vent_: calculated $\overset{\cdot }{V}$O_2_ at respiratory muscles; %$\overset{\cdot }{V}$O_2max_: percentage occupation of $\overset{\cdot }{V}$O_2vent_ to $\overset{\cdot }{V}$O_2max_; P_I__max_: peak inspiratory mouth pressure; P_E__max_: peak expiratory mouth pressure.

a *P* < 0.05 versus NN.

## Discussion

To the best of our knowledge, this is the first study to assess the effects of reduced barometric pressure during acute hypobaric conditions on ventilatory and metabolic responses, as well as the effects on endurance exercise performance during maximal running exercise. The incremental running exercise was performed on separate days under NN (20.9 ± 0.1% O_2_ at 763 ± 5 mmHg), HH (20.9 ± 0.1% O_2_ at 492 ± 1.1 mmHg), and HN (32.2 ± 0.1% O_2_ at 492 ± 1 mmHg) conditions. $\overset{\cdot }{V}$_Emax_ was higher in HN than in NN, although $\overset{\cdot }{V}$O_2max_ did not differ between HN and NN. However, time to exhaustion was longer in HN than in NN. These results suggest that reduced air density associated with acute exposure to 3500 m a.s.l. increases ventilation and improves exercise performance without affecting whole‐body aerobic metabolism.

Our results demonstrate that $\overset{\cdot }{V}$_Emax_ during maximal running is higher in HN than that in NN (Table 1), which could be attributed to reduced air resistance. Higher flow rates through the airways and alveolar branches occurring during maximal exercise often induce turbulent airflow, which is a factor that contributes to increased flow resistance (West 2005). Theoretically, air density would be 0.83 kg m^−3^ at 3500 m a.s.l. and 1.20 kg m^−3^ at sea level, indicating that flow resistance in the airways would be lower in HN than in NN. This ultimately may represent the underlying reason for the higher $\overset{\cdot }{V}$_Emax_ in HN. Our results also demonstrated that $\overset{\cdot }{V}$_E_/$\overset{\cdot }{V}$O_2_ and $\overset{\cdot }{V}$_E_/$\overset{\cdot }{V}$CO_2_ under HN were greater in comparison to those under NN, implying that air‐flow resistance was altered by hypobaria.

Pulmonary ventilation exponentially increases with decreases in ambient partial pressures of O_2_. As previously discussed, our results suggest that air decompression associated with exposure to HH could increase ventilation during exercise. The $\overset{\cdot }{V}$_Emax_ in HH was expected to be higher than that in the other two conditions as a consequence of hypoxia and air decompression. However, we observed that $\overset{\cdot }{V}$_Emax_ in HH was not different from that in NN and HN. The precise reason for this finding remains to be established. However, it may be attributable to the reduced absolute exercise intensity (running speed) at $\overset{\cdot }{V}$O_2max_ in HH relative to the other two conditions. Hence, greater ventilatory drive associated with the combination of hypoxia and air decompression is offset by a lower ventilatory drive associated with lower exercise intensity.

SaO_2_ at maximal running in NN was 91% (Table 1), indicating that our participants developed exercise‐induced arterial hypoxemia. Under the NN condition, any increase in oxygen supply due to increased ventilation appears to have increased SaO_2_ and $\overset{\cdot }{V}$O_2max_. Although $\overset{\cdot }{V}$_Emax_ during maximal running was higher in HN than in NN, neither SaO_2_ nor $\overset{\cdot }{V}$O_2max_ increased (Table 1). In contrast, Powers et al. (1986) reported that among individuals with exercise‐induced arterial hypoxemia under NN, breathing He–O_2_ resulted in increased $\overset{\cdot }{V}$O_2max_ and a 29% increase in $\overset{\cdot }{V}$_Emax_ during intense exercise. We also previously reported that in HH at 2500 m a.s.l., breathing He‐O_2_ increased $\overset{\cdot }{V}$O_2max_ and resulted in a 15.1% increase in $\overset{\cdot }{V}$_Emax_ (Ogawa et al. 2010). In the present study, the lack of effect from increased $\overset{\cdot }{V}$_E_ on SaO_2_ and $\overset{\cdot }{V}$O_2max_ under the HN condition could be due to a relatively smaller increase in $\overset{\cdot }{V}$_Emax_ (6.8%) relative to that experienced under the NN condition (previous studies utilizing He–O_2_ gas showed a greater increase in $\overset{\cdot }{V}$_E_ of 15–29%). Moreover, this study demonstrated that the increase in $\overset{\cdot }{V}$_Emax_ was mainly caused by an increase in *f*_R_ without a measurable increase in VT. This result implies that a large portion of the increase in $\overset{\cdot }{V}$_Emax_ in HN relative to that in NN may have resulted from increased dead space with a minimal increase in alveolar ventilation. In fact, $\overset{\cdot }{V}$_A_ and P_A_O_2_ were not different between HN and NN (Table 1). One might think that the reduced airway resistance associated with hypobaria would reduce turbulent airflow, thereby minimizing physiological dead space; however, this effect, if present, may have been overpowered by the rapid shallow breathing that occurred in HN.

The lack of effect of increased ventilation on $\overset{\cdot }{V}$O_2max_ in HN is in line with the estimations reported in previous studies. Regarding reduced air density, Esposito and Ferretti (1997) demonstrated that when $\overset{\cdot }{V}$_E_ increased with He‐O_2_ breathing, $\overset{\cdot }{V}$O_2max_ increased during He‐O_2_ breathing under hypoxic conditions, while $\overset{\cdot }{V}$O_2max_ did not increase under normoxic conditions. Although the air density in He‐O_2_ is greatly reduced compared to that in 3500 m a.s.l. hypobaria, our results under HN were consistent with the results of their normoxic He‐O_2_ breathing results. Further, as a limitation of $\overset{\cdot }{V}$O_2max_, the ventilatory resistance that limits the flow of O_2_ from the atmosphere to the alveolar sacs could be analyzed using the multifactorial model proposed by di Prampero (2003). According to this model, the resistance imposed on O_2_ flow because of ventilatory resistance decreased by 13% under HN compared to that under NN (data not shown). If the resistance to O_2_ flow is altered, thereby changing $\overset{\cdot }{V}$O_2max_, the fractional limitation to $\overset{\cdot }{V}$O_2max_ imposed by ventilatory resistance to O_2_ flow (F_v_) can be calculated. Ferretti and di Prampero (1995) reported that F_v_ was 5% under NN. The calculated F_v_ in the present study was 4%, which agrees closely with the estimations made by Ferretti and di Prampero (1995), implying that the contribution of $\overset{\cdot }{V}$_E_ was not a limiting factor for $\overset{\cdot }{V}$O_2max_ in NN among the participants in the current study.

Exercise performance based on the time to exhaustion was extended in HN compared with that in NN (Table 1). This implies that reduced airway resistance associated with hypobaric exposure could improve endurance exercise performance. This may be counterintuitive, as $\overset{\cdot }{V}$O_2max_ (aerobic energy supply) did not differ between HN and NN conditions in this study (Table 1). However, similar results were also reported by Marconi et al. (2004) with chronic hypobaric hypoxic exposure (5050 m a.s.l.). We do not know the exact mechanism by which reduced airway resistance under HH conditions improves endurance exercise performance without affecting $\overset{\cdot }{V}$O_2_, but some insights could be gleaned from a previous work. Diaphragmatic fatigue during strenuous ventilation has been shown to increase the activity of sympathetic nerves that innervate muscles (Derchak et al. 2000). This results in reduced active muscle blood flow (Sheel et al. 2001). $\overset{\cdot }{V}$O_2rm_ comprises a significant portion of whole‐body $\overset{\cdot }{V}$O_2_ because of hyperventilation that occurs during intense exercise (Aaron et al. 1992; Vella et al. 2006). Along these lines, Harms et al. reported that unloading the work of respiratory muscles because of inspiratory assistance during intensive exercise results in improved exercise tolerance with a greater distribution of the available cardiac output to active locomotor muscles with no increase in whole‐body $\overset{\cdot }{V}$O_2_ (Harms et al. 1997, 1998, 2000). In our study, P_Imax_ and P_Emax_ were lower and $\overset{\cdot }{V}$_E_ was higher in HN compared with that in NN during the mimic ventilatory tests (Table 2). This suggests that air flow resistance during maximal exercise may be reduced because of reductions in air density associated with hypobaric exposure (3500 m a.s.l.). Further, decreased work during respiration was indirectly supported by our results. We demonstrated that the estimated $\overset{\cdot }{V}$O_2rm_ was lower under HN than under NN and that the estimated distribution of $\overset{\cdot }{V}$O_2rm_ was lower under HN than under NN (Table 2 and Fig. 1). These results suggest that the oxygen supply to active muscles was increased in exchange for reducing the oxygen consumption of the respiratory muscles. Therefore, this may improve exercise performance in HN compared with that in NN.

### Limitations

A limitation of this study was that participants knew the conditions under which they were exercising. We do not know if this might have affected our results and if so, to what extent. We assessed the influence of hypobaria by comparing responses between NN and HN conditions in the absence of hypoxia. Additional studies are required to elucidate whether hypobaria can modulate responses under hypoxic conditions. Our results also may have been different if a different exercise protocol had been employed. Finally, we did not directly assess airway resistance. However, in the present study, we observed lower oral pressure and respiratory muscle $\overset{\cdot }{V}$O_2_ despite the fact that a higher $\overset{\cdot }{V}$_Emax_ was observed under HN compared with NN. Therefore, we believe that airway resistance was substantially reduced with exposure to HH. Moreover, had we employed a cycling model, we might have been able to assess the relationships between $\overset{\cdot }{V}$_E_ and $\overset{\cdot }{V}$O_2_ at a given work rate. This information would be helpful to evaluate whether respiratory efficiency would be altered under hypobaric conditions.

## Conclusion

We found that $\overset{\cdot }{V}$_Emax_ was higher and the time to exhaustion during incremental running was extended under HN compared with that in NN and there was no difference in $\overset{\cdot }{V}$O_2max_. This suggests that reduced air density under the hypobaric condition of 3500 m a.s.l. improved exercise performance without increasing aerobic energy supply, possibly because of a reduced oxygen supply to respiratory muscles and a concomitant increase in oxygen supply to active muscles.

## Conflict of Interest

None.
